# A remarkable new species of *Brunfelsia* (Solanaceae) from the eastern Andes of Central Peru

**DOI:** 10.3897/phytokeys.75.10759

**Published:** 2016-12-01

**Authors:** James G. Graham, John P. Janovec

**Affiliations:** 1 Botany Department, Field Museum, 1400 S. Lake Shore Drive, Chicago, IL USA; 2 Herbario Forestal MOL, Facultad de Ciencias Forestales, Universidad Nacional Agraria La Molina, Lima, Peru

**Keywords:** Brunfelsia, Solanaceae, Peru, Cordillera El Sira, Cordillera Yanachaga

## Abstract

*Brunfelsia cabiesesiana* J. G. Graham, **sp. nov.** (Solanaceae), a new species from montane cloud forests of Ucayali and Pasco Departments, Peru, is described and illustrated. The new species differs from all other members of the genus *Brunfelsia* by its cauline inflorescences. A key to the Peruvian species of *Brunfelsia* is presented.

## Abstract (es)

*Brunfelsia cabiesesiana* J. G. Graham, **sp. nov.** (Solanaceae), descrita e ilustrada aquí, constituye una nueva especie de los bosques nublados de los departamentos de Ucayali y Pasco, Perú. La nueva especie difiere de todas las demás especies de Brunfelsia por sus inflorescencias caulinas. Se presenta aquí también una clave para las especies peruanas de Brunfelsia.

## Introduction

**Figure 1.**
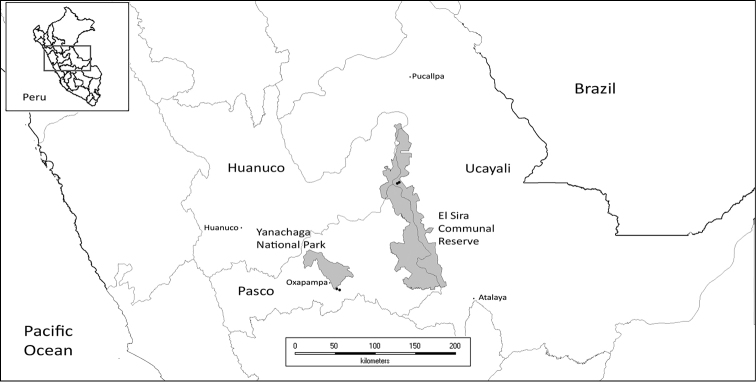
Location of known specimens of *Brunfelsia cabiesesiana*.

During the course of botanical exploration in the Cordillera El Sira (see Figure 1), we encountered an interesting species of *Brunfelsia* with a unique combination of features differing from all other members of the genus. After reviewing specimens of *Brunfelsia* deposited in herbaria at F, HOXA and MOL (Thiers 2016), and digitized specimens available at JSTOR Global Plants (http://plants.jstor.org/), we describe a unique new species. Illustrations, photographs, a discussion of affinities, and a key to Peruvian species of *Brunfelsia* is presented.

The Solanaceae (nightshade family) are widely distributed across the globe, including ca. 96 genera and approximately 2800 species of herbs, shrubs, trees, vines, lianas and epiphytes, with its greatest concentration of species found in the New World. The most recent taxonomic classification by Barbosa et al. (2016) recognizes five subfamilies and 14 tribes.

*Brunfelsia* is a neotropical genus known from the Caribbean and South America as far north as Panama. Its taxonomic history has been well documented by Plowman (1974, 1998), beginning with a short description, diagnosis and illustration by Plumier (1703), named (and misspelled) *Brunsfelsia* in honor of German herbalist Otto Brunfels. Linnaeus included *Brunfelsia* in the second edition of Genera Plantarum (1742), based on Plumier’s description; the valid publication of the genus dates from 1753, when he published *Brunfelsia americana* in Species Plantarum. Tribal placement of *Brunfelsia* has fluctuated. Beginning with Bentham’s (1835) assignment of the genus to tribe Salpiglossideae, reassignment to tribe Francisceae (Don 1837), back to Salpiglossideae again (Endlicher 1839, Bentham 1846), until Miers (1849) placed the genus in tribe Brunfelsiae. Bentham and Hooker (1873) moved it back to Salpiglossideae, where it was conserved by Baillon (1888), van Wettstein (1895), Baehni (1946), and Plowman (1974). Hunziker (2001) placed *Brunfelsia* as the sole genus in Tribe Fracisceae, and Olmstead et al. (2008), considering chloroplast DNA sequences, placed the genus in tribe Petunieae. The most recent taxonomic treatment of Solanaceae by Barbosa et al. (2016) places the genus with those taxa lacking clear relationships, i.e. *Incertae sedis*. It is interesting to note that the Petuniae of Olmsead et al. (2008), and Plowman’s Salpiglossideae share nearly half of their genera (four of nine- *Brunfelsia* L., *Hunzikeria* D’Arcy, *Leptoglossis* Benth. and *Plowmania* Hunz. & Subils).

Plowman (1974, 1998) considered *Brunfelsia* to be a distinct genus, not readily confused with other genera. Its closest relatives were considered to be the genera *Browallia* and *Streptosolon*; these are easily distinguished because the the woody habit and indehiscent capsules of *Brunfelsia* are not present in *Browallia*, nor is the twisted corolla tube and bright red-orange limb of *Streptosolon* found in *Brunfelsia*.

Plowman (1978, 1998) recognized three subgeneric sections, Brunfelsia sect. Guianensis Plowman; Brunfelsia sect. Franciscea (Pohl) Griseb., and Brunfelsia sect. Brunfelsia L., based on differences in floral morphology and distinct and mostly allopatric areas of distribution. This classification has been tested by recent molecular work of Filipowicz and Renner (2012). Their sequence data supported two clades, an Antillean clade consisting of all Brunfelsia sect. Brunfelsia and a second clade consisting of Brunfelsia sect. Guianensis and Plowman’s Brunfelsia sect. Franciscea. Their molecular work resulted in the description of a new species (*Brunfelsia plowmaniana* N. Filipowicz & M. Nee) (Filipowicz et al. 2012), recovered from the Bolivian/Argentine members of *Brunfelsia uniflora* (Pohl) D.Don.

All *Brunfelsia* species reported from Peru (i.e., *Brunfelsia chiricaspi* Plowman, *Brunfelsia grandiflora* D.Don, and *Brunfelsia mire* Monach.) have showy violet flowers that fade in color as they mature, each with a distinctive white eye at the throat. No fewer than five species are recorded from Andean regions of South America and ours is readily distinguishable from all members of the genus, including its nearest neighbors, by the presence of cauline inflorescences.

## Taxonomic treatment

This new species is distinguished by its unique cauline inflorescence not encountered in any other member of the genus. A key to distinguish other known Peruvian species is provided.

### Key to the Peruvian species of *Brunfelsia* (adapted from Plowman 1998)

**Table:** 

1	Inflorescences terminal, subterminal or axillary	2
–	Inflorescences cauline (borne along the main trunk or on tertiary vertical branchlets)	***Brunfelsia cabiesesiana***
2	Corolla lobes spreading at anthesis; leaves less than 8 cm wide	3
–	Corolla lobes deflexed at anthesis; leaves greater than 8 cm wide	***Brunfelsia chiricaspi***
3	Leaves more or less two ranked, scattered along branchlets, with 5-9 lateral nerves; inflorescences lax, often short-branched; corolla tube 15-40 mm long	***Brunfelsia grandiflora***
–	Leaves crowded towards apex of stem, subverticillate, to about six per whorl, with 8-13 lateral nerves; inflorescences dense, capituliform; corolla tube 25-38 mm long	***Brunfelsia mire***

### Brunfelsia cabiesesiana

Taxon classificationPlantaeSolanalesSolanaceae

J.G.Graham sp. nov.

urn:lsid:ipni.org:names:77158825-1

**Figure 2.**
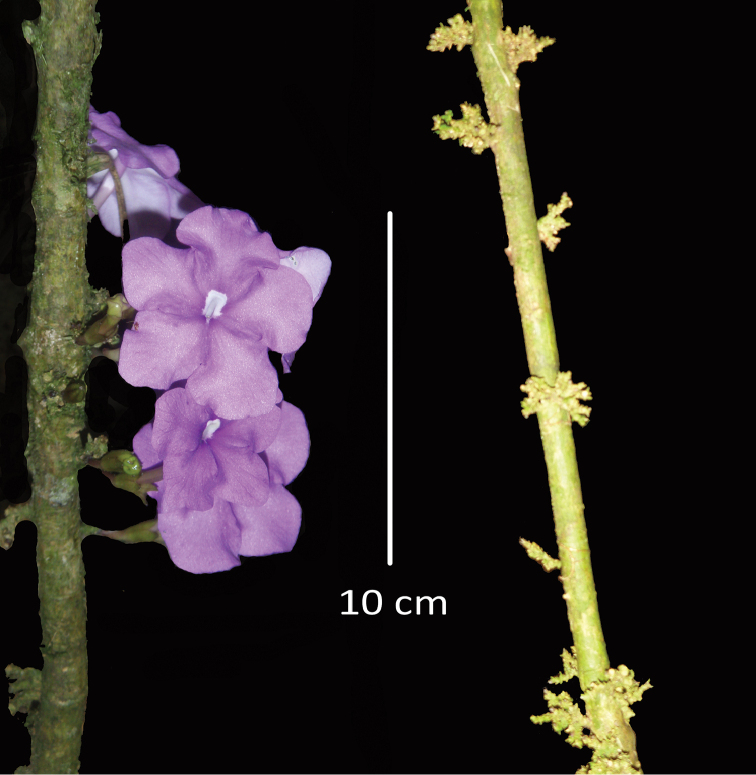
Inflorescences of *Brunfelsia cabiesesiana*. Left, cauline corymbiform inflorescences showing limb of corolla at anthesis. Right, stem with bracteate inflorescence branches.

**Figure 3.**
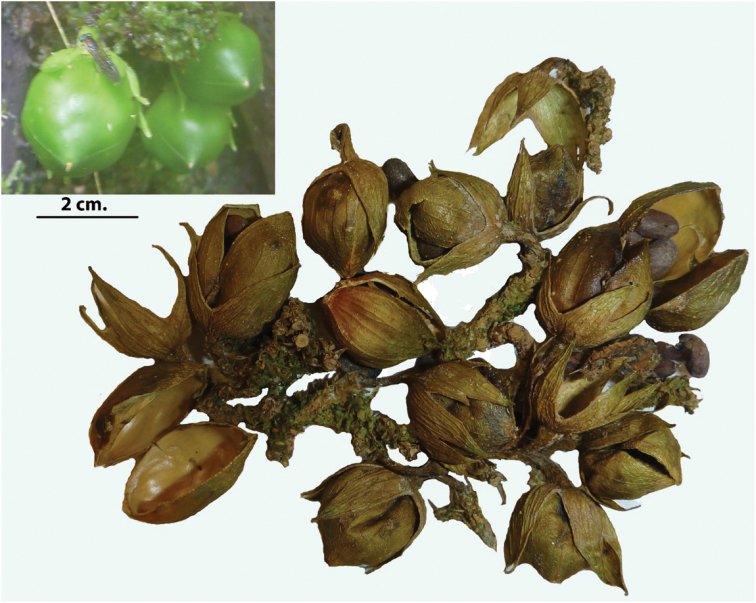
Fruits of *Brunfelsia cabiesesiana*. Below, dried, dehiscent capsules with seeds. Above left, developing fruits.

**Figure 4.**
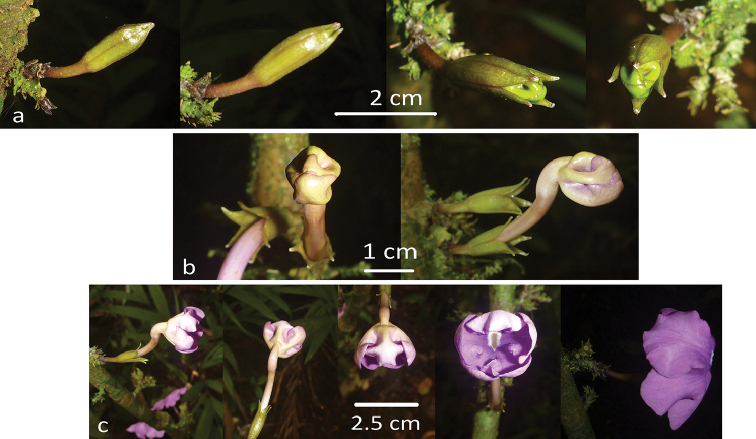
Estivation of *Brunfelsia cabiesesiana*. **A** calyces in bud. (day 1–3) **B** emerging flower. (day 4–5) **C** petals unfold (day 6–7).

**Figure 5.**
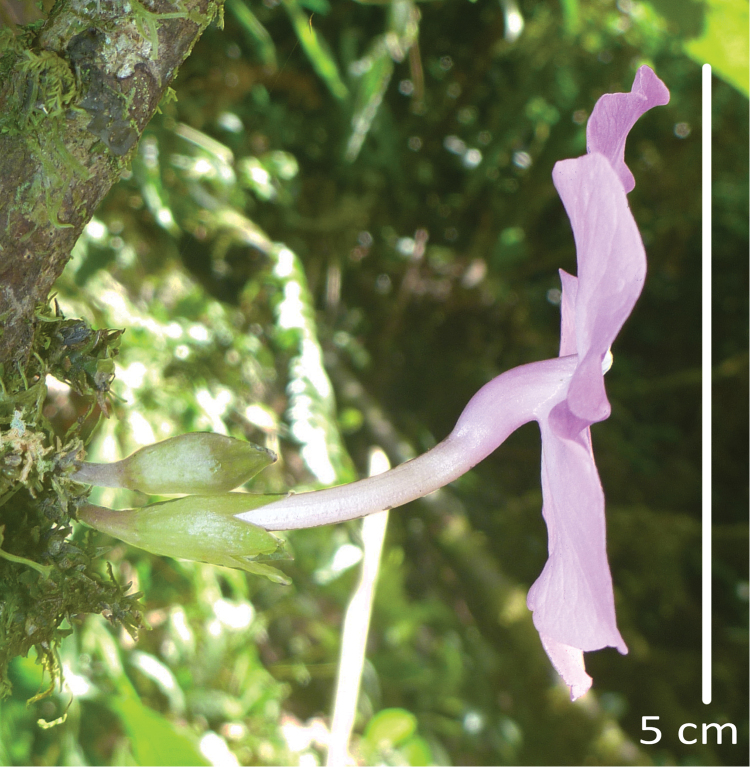
Profile of a flower of *Brunfelsia cabiesesiana* at anthesis.

**Figure 6.**
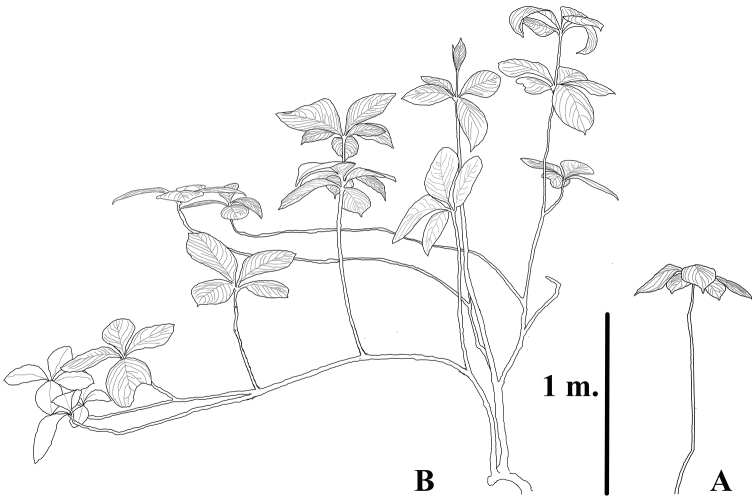
Two habit forms of *Brunfelsia cabiesesiana* found in the El Sira Mountains (drawing by JGG).

Figures 2 , 3 , 4 , 5 , 6

#### Type.

PERU. Ucayali: Prov. Coronel Portillo, Dist. Iparia, Reserva Comunal El Sira, 1500 m, 9°27.8'S, 74°33.5'W, 24 Oct 2007, *J. G. Graham 5970* (holotype: MOL; isotypes: F, NY).

#### Diagnosis.

*Brunfelsia cabiesesiana* distinguitur ab omnibus aliis speciebus *Brunfelsia* possidendo cauliflorus inflorescentiis.

#### Description.

Pachycaulescent shrub to few-branched, sprawling small tree to 3 m. *Trunk* solitary, terete, to 5 cm in diameter near base. *Bark* brownish-gray and rough at stem base, becoming dark green and smooth on upper stem; glabrous. *Branches* lacking, or, if present, terete, to 2 cm diameter, tending to arch over with age, with vertically ascending branchlets. *Leaves* crowded toward apex of stem, subverticillate, up to seven per whorl, simple, often in terminal whorls on single stem, occasionally in multiple whorls 20–30 cm apart; petioles sub-terete, often canaliculate above, up to 1cm long, 3–5 mm wide, brownish when dry, blades elliptic to broadly obovate, 15–35 cm long, 6–15 cm wide, glabrous, dull, dark green above, pale green beneath, young leaves purplish, smooth, subcoriaceous, glabrous, the base narrowly decurrent, the apex cuspidate to lightly acuminate, the margins entire; the midvein prominent below, the secondary veins 6–8-nerved, spaced up to 2 cm apart, arcuate-ascending, with light collective vein on margin, the tertiary venation reticulate. *Inflorescences* cauline, corymbiform, flowering branches stunted, woody at base, persistent, leafless, densely bracteate, to 3 cm long, with 1–7 branchlets up to 5 mm long, few flowered, usually only 1 flower per branchlet. *Bracts* spirally arranged, lanceolate, lightly keeled below, 0.7–1.3 mm long, tan to brown, lightly pubescent at base and along margins. *Flowers* showy violet fading with age, with 5-angled white spot at mouth. *Pedicels* 4–10 mm long, slender, 1.5–2 mm in diameter, glabrous. *Calyx* tubular-campanulate, weakly inflated, 2–2.3 cm long, 6–8 mm in diameter, ellipsoid to ovoid in bud, yellow-green to green, lightly punctate, firmly membranaceous, connate at base, 5-lobed at apex, the lobes subequal, ovate-lanceolate, 4–8 mm long, acute to acuminate and glandular at apex; calyx to 2.2 cm in fruit, coriaceous, smooth, partially enclosing the fruit, calyx and pedicel often with raised lenticels at maturity. *Corolla tube* terete, curved and inflated slightly at apex, then constricted at throat, gradually widening from base, 2.5–4 cm long, 2 mm diameter at base, to 5 mm diameter at apex, glabrous; estivation quincuncial and imbricate, the limb spreading to 5.5 cm diameter, the lobes rounded, subequal, uppermost slightly larger, subtruncate to rounded at apex, overlapping at sides, narrowing lightly at base. *Stamens* four, in two pairs, included in upper portion of corolla tube; filaments ligulate, curved at apex, 2–3 mm long; anthers reniform, to 2 mm long. Ovary ovoid-conical, 2–3 mm long, glandular at base; style slender, curved and thickened at apex, 3–3.5 cm long; stigma weakly/briefly bifid, lobes equal, oval, 1–2 mm long, 8–10 mm in diameter. *Fruit* a capsule, globose to ovoid, 1.5–2 cm long, 1.2–1.8 cm wide, partially enclosed by accrescent calyx, slightly acute at apex with conspicuous scar where corolla tube was attached, medial septicidal suture present, not dehiscing along suture, dark green when fresh, light brown and lightly veined when dry, smooth, glabrous, pericarp thin walled, 1–2 mm thick, drying crustaceous, exocarp coriaceous, lenticelate. *Seeds* 10–20 per fruit, oblong-ellipsoid, 5–7 mm long, ca. 3 mm in diameter, dark reddish-brown with brilliant prismatic reflection, reticulate pitted, glabrous.

#### Habitat and ecology.

Known from central Peru in the Departments of Ucayali and Pasco where it is of extremely limited distribution but locally abundant at the type locality in the El Sira Communal Reserve. This understory species inhabits rocky slopes and ridge tops in cloud forests on the eastern slopes of the Cordillera El Sira, between 1100–1600 m, and has been found as a rare element in cloud forests on the northwestern slopes of the Cordillera Yanachaga, at ca. 2300 m.

#### Phenology.

Flowering in *Brunfelsia cabiesesiana* appears to be photomorphogenic in nature, associated with highest annual light intensities. Flowering observed in the El Sira populations is closely associated with the dry season, from August to October. Fruits appear to mature relatively slowly and are persistent, having been found green on the plant two or more months following anthesis.

#### Etymology.

The species epithet honors Dr. Fernando Cabieses Molina, noted neurosurgeon, ethno-pharmacologist, author and educator. Dr. Cabieses was cofounder of the *Museo de la Nación* of Peru and founding rector of the Universidad Científica del Sur. He served as director of the Peruvian Museum of Health Sciences and the Peruvian National Institute of Traditional Medicine. Dr. Cabieses had profound interest in tropical biodiversity -both its history and utilization- and he was a tireless supporter of biodiversity conservation efforts in Peru.

#### Conservation status.

This species is of extremely limited distribution (see Figure 1), although it appears to be locally abundant as evidenced by preliminary density studies along an elevational transect near the type locality, where 18 individuals were recorded in 2000 sq. m area. Three of these had reached maturity, as evidenced by stunted, persistent inflorescence branches (see Figure 2). This species appears to be extremely rare in the Cordillera Yanachaga, ca. 125 km SW from the type location and nearly 800 m higher in elevation.

Given the extremely limited known area of occupancy of *Brunfelsia cabiesesiana*, and the fragmented nature of the occurrence of the two known subpopulations in Pasco and Ucayali, we estimate the Pasco subpopulation to be critically endangered and the Ucayali population to be endangered, using International Union for the Conservation of Nature Red Book guidelines (IUCN 2012). Both of the Pasco collections were located in fragmented forests near to roads. Given that anthropogenic activity in this region continues to expand, and that only two collections have ever been made, it is considered to face an extremely high risk of extinction in the wild. The Ucayali subpopulation, with the benefit of larger species densities, as well as a more favorable location inside a reserved zone, faces less threat of extinction.

#### Specimens examined.

**PERU. Ucayali**: Dist. Iparia, Reserva Comunal El Sira, 9°28'S, 74°34'W, 1550 m, 24 Oct 2007, *J. G. Graham 4968* (F, MOL, NY); **Pasco**: Dist. Oxapampa, 10°37'S, 75°20'W, 2100 m, Villa Rica - Oxapampa, 4 Jan 1984, *R. Foster et al. 7788* (F); Dist. Oxapampa, 10°30'S, 75°20'W, 4 Aug 2009, forest remnant at the edge of a road, *R. Vasquez et al. 36203* (HOXA).

## Discussion

*Brunfelsia cabiesesiana* sp. nov. has a combination of characters that clearly separate it from other species of *Brunfelsia*: its strictly cauliflorous habit distinguish it from all other members of the genus, including the verticillate-leaved (*Brunfelsia mire* Monachino, *Brunfelsia hydrangeiformis* (Pohl) Benth.) members of the genus, as well as those with one central trunk (*Brunfelsia densifolia* Krug & Urb., *Brunfelsia mire*, *Brunfelsia chiricaspi* Plowman).

There is a clear divergence in elevational range between the Yanachaga and El Sira populations of *Brunfelsia cabiesesiana*. A similar elevational-displacement phenomenon has been recorded for other organisms in the Cordillera El Sira. Terborgh and Weske (1975) noted a downward-displacement in elevation for species ranges of birds in the Cordillera El Sira, compared with the Cordillera Vilcabamba, with displacements of approximately 800 m. A similar pattern of elevational range displacement between the Cordilleras Yanachaga and El Sira, in both vascular plants and bryophytes, has been observed during our own field research.

## Supplementary Material

XML Treatment for Brunfelsia cabiesesiana
